# Incidence of bloodstream infections due to multidrug-resistant pathogens in ordinary wards and intensive care units before and during the COVID-19 pandemic: a real-life, retrospective observational study

**DOI:** 10.1007/s15010-023-02000-3

**Published:** 2023-03-03

**Authors:** Francesco Vladimiro Segala, Pia Clara Pafundi, Carlotta Masciocchi, Barbara Fiori, Eleonora Taddei, Laura Antenucci, Giulia De Angelis, Silvia Guerriero, Roberta Pastorino, Andrea Damiani, Brunella Posteraro, Maurizio Sanguinetti, Gennaro De Pascale, Massimo Fantoni, Rita Murri

**Affiliations:** 1 Dipartimento di Sicurezza e Bioetica, Sezione di Malattie Infettive, Università Cattolica del Sacro Cuore, Rome, Italy; 2 Facility of Epidemiology and Biostatistics, Gemelli Generator, Fondazione Policlinico Universitario A. Gemelli IRCCS, Rome, Italy; 3 Real World Data Facility, Gemelli Generator, Fondazione Policlinico Universitario A. Gemelli IRCCS, Rome, Italy; 4 Dipartimento di Scienze di Laboratorio e Infettivologiche, Fondazione Policlinico Universitario A. Gemelli IRCCS, Rome, Italy; 5 Dipartimento di Scienze Biotecnologiche di Base, Cliniche Intensivologiche e Perioperatorie, Università Cattolica del Sacro Cuore, Rome, Italy; 6 Department of Anesthesia and Intensive Care, Agostino Gemelli Hospital, Università Cattolica del Sacro Cuore, Rome, Italy

**Keywords:** Antimicrobial resistance, COVID, Acinetobacter, MRSA, Intensive care unit

## Abstract

**Purpose:**

SARS-COV-2 pandemic led to antibiotic overprescription and unprecedented stress on healthcare systems worldwide. Knowing the comparative incident risk of bloodstream infection due to multidrug-resistant pathogens in COVID ordinary wards and intensive care-units may give insights into the impact of COVID-19 on antimicrobial resistance.

**Methods:**

Single-center observational data extracted from a computerized dataset were used to identify all patients who underwent blood cultures from January 1, 2018 to May 15, 2021. Pathogen-specific incidence rates were compared according to the time of admission, patient’s COVID status and ward type.

**Results:**

Among 14,884 patients for whom at least one blood culture was obtained, a total of 2534 were diagnosed with HA-BSI. Compared to both pre-pandemic and COVID-negative wards, HA-BSI due to *S. aureus* and *Acinetobacter* spp*.* (respectively 0.3 [95% CI 0.21–0.32] and 0.11 [0.08–0.16] new infections per 100 patient-days) showed significantly higher incidence rates, peaking in the COVID-ICU setting. Conversely, *E. coli* incident risk was 48% lower in COVID-positive vs COVID-negative settings (IRR 0.53 [0.34–0.77]). Among COVID + patients, 48% (*n* = 38/79) of *S. aureus* isolates were resistant to methicillin and 40% (*n* = 10/25) of *K. pneumoniae* isolates were resistant to carbapenems.

**Conclusions:**

The data presented here indicate that the spectrum of pathogens causing BSI in ordinary wards and intensive care units varied during the pandemic, with the greatest shift experienced by COVID-ICUs. Antimicrobial resistance of selected high-priority bacteria was high in COVID positive settings.

**Supplementary Information:**

The online version contains supplementary material available at 10.1007/s15010-023-02000-3.

## Purpose

Antibiotic resistance (AMR) has emerged, worldwide, as one of the greatest threats facing public health in the twenty-first century. It occurs when organisms become less sensitive to the action of a given antimicrobial and, globally, it caused at least 1.27 million deaths and it was linked to almost 5 million deaths only in 2019 [1].

On the basis of a multicriteria analysis that included resistance trends, expected availability of effective drugs and overall healthcare and community burden, the World Health Organization (WHO) identified the following list of high-priority resistant pathogens: carbapenem-resistant *Acinetobacter baumannii* (CRAB), carbapenem-resistant *Pseudomonas aeruginosa* (CRPA), carbapenem-resistant and third generation cephalosporin-resistant Enterobacteriaceae (CRE and 3GCephRE), vancomycin-resistant *Enterococcus faecium* (VRE) and methicillin-resistant *Staphylococcus aureus* (MRSA) [2]. Those bacteria largely overlap with the most commonly resistant pathogens isolated in the European region in 2020 [3].

In this scenario, the pandemic caused by severe acute respiratory syndrome coronavirus 2 (SARS-CoV2) disrupted healthcare systems and practices all over the world. Challenges experienced by hospitals include staffing shortages, unprecedentedly high hospitalization rates and supply constraints that may have led, in turn, to changes in infection control practices [4] and antibiotic overuse [5]. This combination has been described by the Centre for Disease Control and Prevention (CDC) as the “perfect storm” for the spread of healthcare-associated, antibiotic-resistant infections [6]. Preliminary analyses, reports and case series described sporadic outbreaks of drug-resistant pathogens in COVID-19 ICUs, especially carbapenem-resistant *A. baumannii* (CRAB) [7], methicillin-resistant *S. aureus* (MRSA), carbapenem-resistant *K. pneumoniae* (KPC-Kp), and vancomycin-resistant *Enterococcus* spp. (VRE) [8]. Nevertheless, despite the concerns, clear evidence is still lacking about the exact incidence of drug-resistant infections in COVID settings, both in general wards and intensive-care units (ICUs). Furthermore, available studies exploring the issue of drug-resistant nosocomial infections during the COVID period are often missing a control group, focus mainly on COVID-ICUs and do not provide information on incidence rates. Little is known about the incidence of resistant infections in non-ICU COVID setting. In addition, while outbreaks resolve after the surge, the medium and long-term impact of COVID-related healthcare disruption on AMR is unknown.

The present study aims to describe the longitudinal trends in the incidence of hospital-acquired bloodstream infections (BSI) due to high-priority pathogens during and before the advent of the COVID-19 pandemic.

## Methods

### Setting and study design

We conducted this real-life, retrospective cohort study at the University Hospital “Policlinico Agostino Gemelli” IRCCS, Rome, Italy. We extracted clinical and microbiological data from a computerized dataset by developing a standardized, ontology-based Data Mart hosted by Gemelli Generator infrastructure [9].

The construction of the Data Mart was based on the ontology defined by an interdisciplinary team composed of infectious disease specialists and microbiologists, who identified an extensive list of variables to be considered for the study. Study Data Mart was developed using the SAS Institute software analysis tool and the SAS® Vyia® environment.

All patients hospitalized during the study period for whom at least one blood culture was performed were eligible to be included in the Data Mart. Only the first BSI episode for each patient was considered. The presence of BSI was defined as the growth of a clinically important pathogen in at least one blood culture or, in the case of typical blood culture contaminants, as the growth of the same microorganism in multiple samples belonging to the same blood culture set. Blood cultures yielding more than one pathogen were defined as polymicrobial. Microorganisms growing only from the central venous catheter (CVC) samples—and not in paired peripheral access samples—were considered contaminants and thus excluded from the study. Also, all positive blood cultures that were requested within the first 48 h from admission were defined as community-acquired BSIs and excluded from the study. For each positive blood culture, data about the ward type (i.e., general wards or ICU) where the sample was obtained were extracted. BSI were then labelled as ICU-related if cultures were performed after at least 48 h from ICU admission.

All enrolled patients were divided into two historical cohorts: patients hospitalized during SARS-CoV2 pandemic period (i.e. from March 01, 2020 till May 15, 2021) and patients admitted prior to the pandemic (i.e. from December 16, 2018 to February 29, 2020). To analyse populations as much homogeneous as possible, we selected equal periods, i.e. 14.5 months. Patients admitted during the pandemic were further stratified on the basis of their COVID status, and patients who resulted positive for at least one polymerase chain reaction (PCR) COVID-19 nasopharyngeal swab test were diagnosed with SARS-CoV2 infection and were included in the COVID positive cohort.

**Fig. 1:**
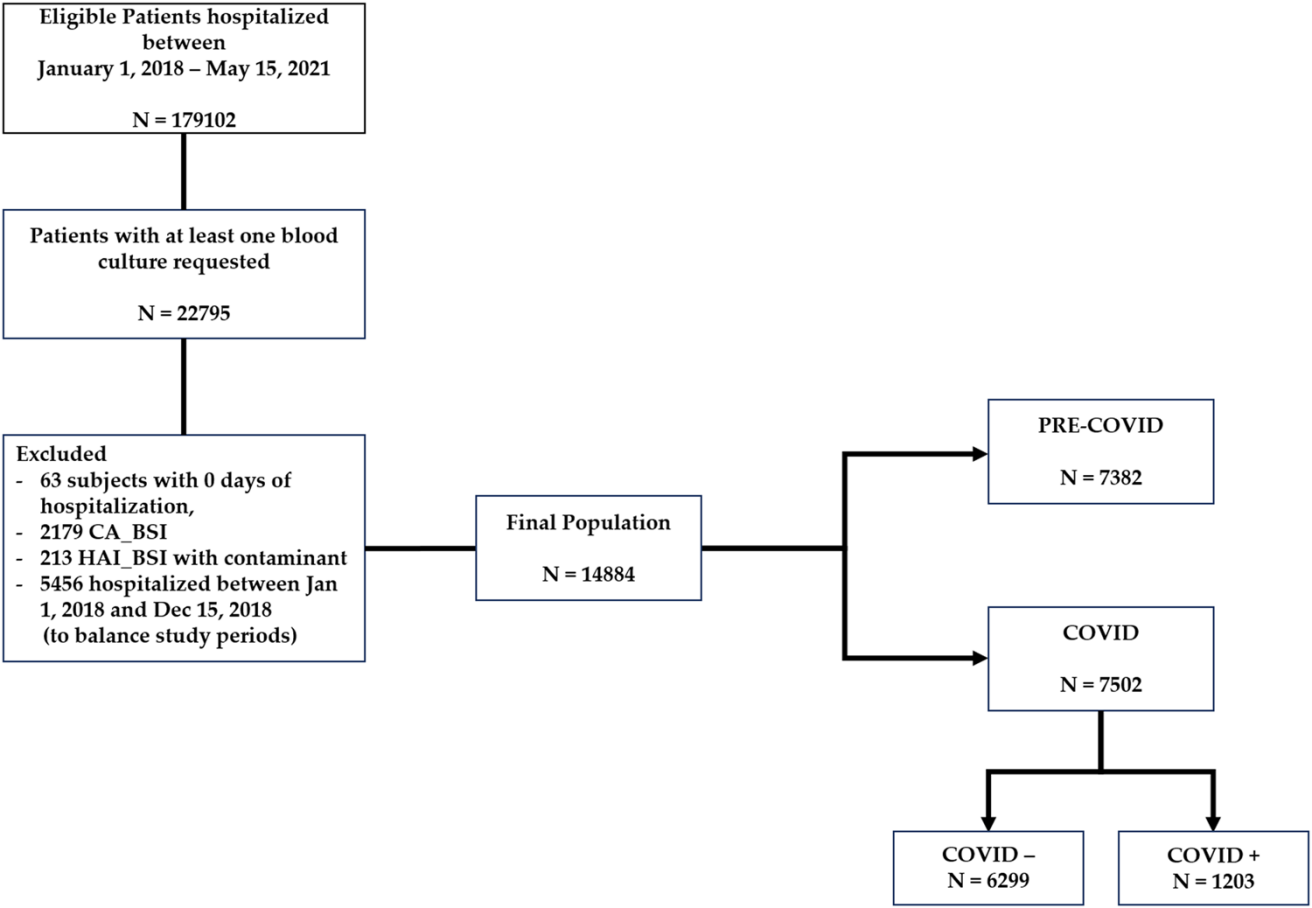
Flow-chart of the study. *CA_BSI* community-acquired bloodstream infection, *HAI_BSI* hospital-acquired bloodstream infection

The flow chart of the study is depicted in Fig. 1.

### Statistical analysis

All variables included in the study were first analysed by descriptive statistic techniques. Data were expressed as absolute and percentage frequencies. Between-group differences were tested either by the Chi-square test or Fisher’s exact test, as appropriate. Differences among patients’ subgroups (i.e., pre-COVID, COVID positive and COVID negative), both overall and pairwise, were assessed by the Fisher exact test (with Freeman-Halton’s extension, when appropriate) or by the Chi-square test. Incidence rates (IR) were computed on the overall population and layered by ward types, i.e., ordinary wards (OW) or intensive care unit (ICU), stratified for study period subgroup (namely pre-pandemic, COVID positive and COVID-negative cohorts). The overall incidence of hospital-acquired bloodstream infections (HAI-BSI) and of each specie was calculated by univariable Poisson regression and defined as the number of events per 100 patient-days of hospitalization.

We further investigated the incidence trends during the pandemic period as compared to pre-pandemic period, as well as differences between COVID-positive and COVID-negative cohorts, on isolates incidence per day of hospitalization. As such, incidence rate ratios (IRR) for HAI-BSIs and specific microorganisms were evaluated by a segmented Poisson regression to model trends over time. Incidence rate comparisons (i.e., IRR and 95% confidence intervals) were further represented by forest plot diagrams, whilst bar plot diagrams describe specific microorganisms’ prevalence across the three subgroups. A *p* value < 0.05 has been considered as statistically significant. The analysis is compliant with the STROBE guidelines for observational studies [10]. All analyses were conducted using R software version 4.2.1 (CRAN ®, R Core 2022) [11].

## Results

From December 2018 to May 2021, 2534 patients out of 14,884 who received at least one blood culture were diagnosed with BSI. Among them, 357 BSIs were polymicrobial. Once performed a blood culture, patients included in the COVID-positive cohort had an overall risk of developing BSI of, respectively, 0.76 (95%CI 0.65–0.89) and 1.16 (95%CI 0.97–1.39) new infections per 100 patient per day in the COVID OW and COVID ICUs (see supplement).

**Fig. 2:**
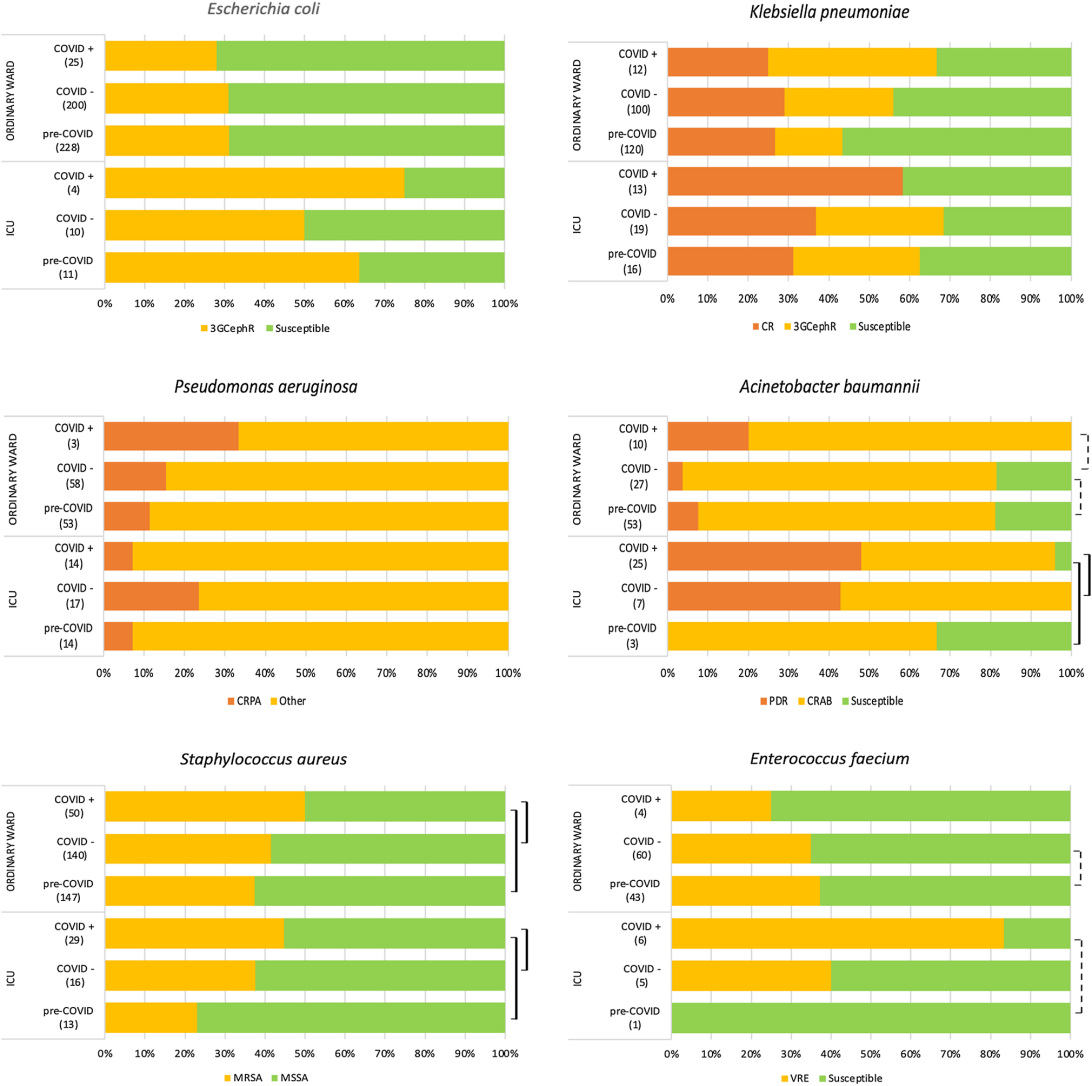
Resistance patterns per study cohort and ward type. The diagrams show the frequencies of selected resistance phenotypes stratified forward type (in brackets the total number of isolates per specie per period). Vertical lines indicate overall and pairwise comparisons according to the studysubgroups (dotted line: *p* < 0.05; continuous line: *p* < 0.001). *CRAB* carbapenem-resistant *A. baumannii*, *CR* carbapenem resistant, *CRPA* carbapenem-resistant *P. aeruginosa*; *MRSA* methicillin-resistant *S. aureus*, *MSSA* methicillin-sensitive *S. aureus*, Other: resistant to at least one antimicrobial class, *PDR* pandrug resistant (limited to tested antimicrobials), *VRE* vancomycin-resistant *E. faecium*; Further information is reported on Supplementary Tables S1 and S2 (web-only)

**Fig. 3:**
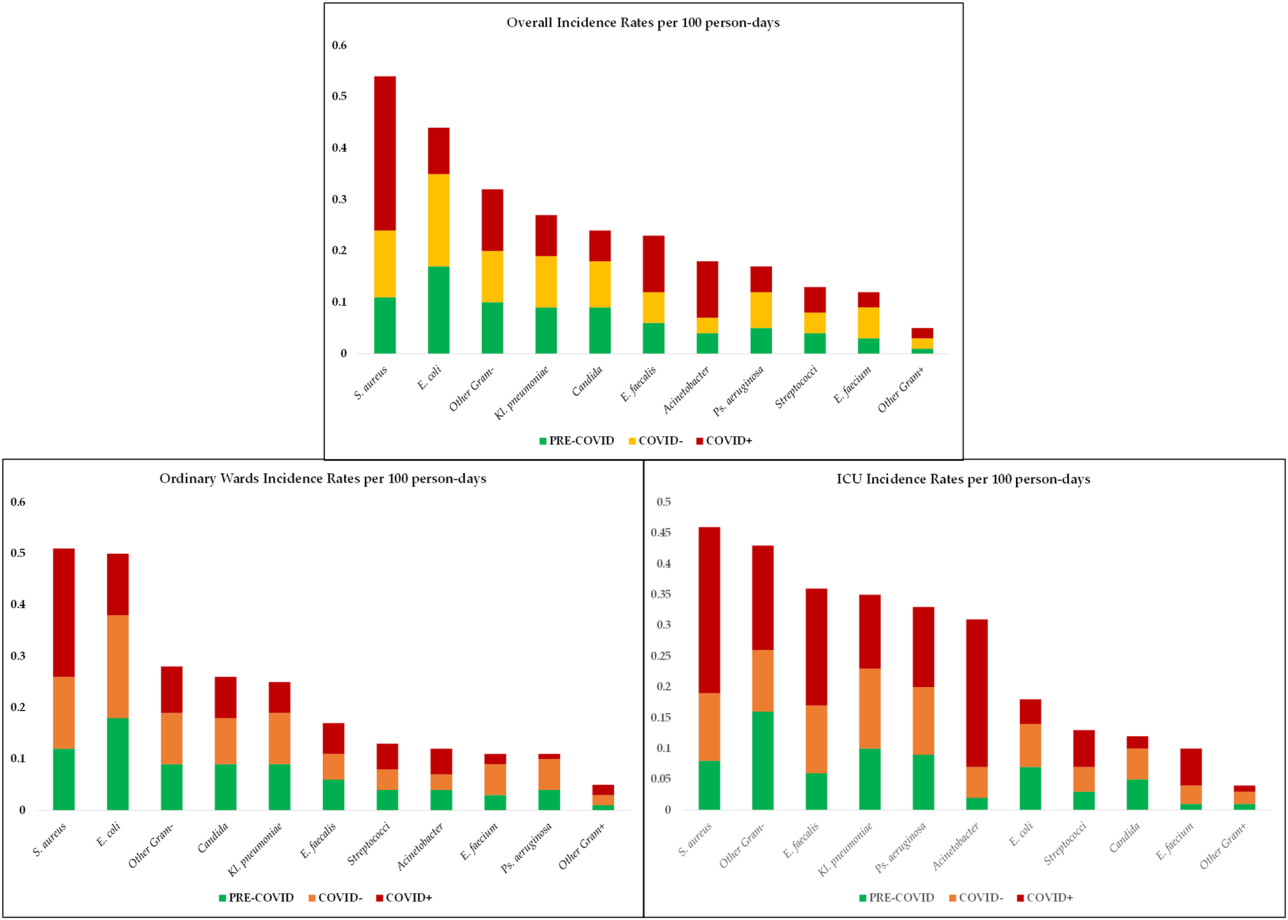
Pathogen-specific incidence rates per ward type. Incidence Rates per 100 persons-days of hospitalization, stratified for study subgroup, i.e. pre-COVID (green, *n* = 7382), COVID negative (orange/yellow, *n* = 6299) and COVID positive (red, *n* = 1203), both overall (upper panel) and layered as ordinary wards (left panel) and ICU (right panel). *ICU* Intensive Care Unit

As shown in Table 1, *E. coli* was the most frequently isolated pathogen, accounting for 478/2534 episodes (18.9%), followed by *S. aureus* (*n* = 395; 15.6%), *K. pneumoniae* (*n* = 280; 11.0%), *P. aeruginosa* (*n* = 159; 6.3%) and *E. faecium* (*n* = 119; 4.7%). Pathogen-specific resistant phenotypes and incidence rates are reported in Figs. 2 and 3. Overall, *E. coli*, *Acinetobacter* spp., *S. aureus*, *E. faecium* and *E. faecalis* frequencies differed significantly across the study periods. Pathogen-specific frequencies in ordinary wards and intensive care units are reported in supplementary Tables S1 and S2 (see supplement). Resistance phenotypes used for the construction of the Data Mart are listed in Table S3.

**Table 1:** Spectrum of microorganisms causing BSI and selected resistance phenotypes stratified by study cohort

	Isolates (percent of phenotypes per species) from											
	COVID period											
	Total BSIs		pre-COVID		COVID neg		COVID pos		*p*^overall^	p*	p**	p***
	No. (%)	%	No. (%)	%	No. (%)	%	No. (%)	%				
Gram-negative bacteria	**1339**	**52.8**	**643**	**55.1**	**554**	**52.5**	**142**	**45.5**				
*Enterobacteriaceae*	758	29.9	375	32.1	329	31.2	54	17.3				
*Escherichia coli*	478	18.9	239	20.5	210	19.9	29	9.3	0.246	0.753	0.126	*0.095*
3GCeph resistant	155 (32.4)		78 (32.6)		67 (31.9)		10 (34.5)		0.958	0.869	0.841	0.781
*Klebsiella pneumoniae*	280	11.0	136	11.6	119	11.3	25	8.0	0.854	0.840	0.576	0.662
3GCeph resistant	63 (22.5)		25 (18.4)		33 (27.7)		5 (20.0)		0.194	*0.076*	0.849	0.425
Carbapenem resistant	83 (29.6)		37 (27.2)		36 (30.2)		10 (40.0)		0.429	0.591	0.196	0.342
Nonfermenting gram-negative bacilli	284	11.2	123	10.5	109	10.3	52	16.7				
*Acinetobacter* spp.	125	4.9	56	4.8	34	3.2	35	11.2	** < 0.001**	0.115	** < 0.001**	** < 0.001**
Carbapenem resistant	86 (82.6)		41 (92.9)		25 (88.2)		20 (60)		0.215	0.974	**0.113**	0.153
XDR	22 (17.6)		4 (7.1)		4 (11.8)		14 (40.0)		** < 0.001**	0.455	** < 0.001**	**0.008**
*Pseudomonas aeruginosa*	159	6.3	67	5.7	75	7.1	17	5.4	0.132	0.103	*0.099*	0.521
Aminoglycoside resistant	10 (6.3)		5 (7.5)		5 (6.7)		0		0.518	0.853	0.245	0.274
3GCeph resistant	27 (17.0)		10 (14.9)		15 (20.0)		2 (11.8)		0.602	0.428	0.739	0.430
Fluoroquinolone resistant	26 (16.3)		11 (16.4)		12 (16.0)		3 (17.6)		0.986	0.946	0.903	0.868
Piperacillin/tazobactam resistant	38 (23.9)		16 (23.9)		19 (25.3)		3 (17.6)		0.798	0.841	0.583	0.502
Carbapenem resistant	22 (13.8)		7 (10.4)		13 (17.3)		2 (11.8)		0.478	0.239	0.875	0.575
Other Gram negatives^§^	297	11.7	145	12.4	116	11.0	36	11.5	**0.032**	0.601	**0.021**	**0.009**
Gram-positive cocci	**1131**	**44.6**	**499**	**42.8**	**469**	**44.4**	**163**	**52.2**				
*Staphylococcus. aureus*	395	15.6	160	13.7	156	14.8	79	25.3	** < 0.001**	0.230	** < 0.001**	** < 0.001**
MRSA	160 (40.5)		58 (36.2)		64 (41.0)		38 (48.1)		0.211	0.383	*0.079*	0.301
*Staphylococcus coagulase negative*	32	1.3	16	1.4	14	1.3	2	0.6	0.928	0.945	0.723	0.700
*Enterococcus faecium*	119	4.7	44	3.8	65	6.2	10	3.2	**0.017**	**0.004**	0.339	0.522
VRE	45 (37.8)		16 (22.7)		23 (35.4)		6 (60)		0.317	0.917	0.170	0.137
*Enterococcus. faecalis*	186	7.3	82	7.0	71	6.7	33	10.6	** < 0.001**	0.928	** < 0.001**	** < 0.001**
*Streptococcus spp.*	113	4.5	53	4.5	44	4.2	16	5.1	*0.059*	0.893	**0.027**	**0.024**
Other Gram Positives	45	1.8	20	1.7	20	1.9	5	1.6	0.670	0.615	0.388	0.588
Fungi												
*Candida* spp.	241	9.5	124	10.6	99	9.4	18	5.8	0.830	0.619	0.644	0.847
Anaerobic bacteria	64	2.5	25	2.1	32	3.0	7	2.2	0.225	0.125	0.199	0.744
Total BSIs	**2534**		**1167**		**1055**		**312**					
Negative blood cultures	**12,350 (20.5)**		**6215 (18.7)**		**5244 (20.1)**		**891 (35)**					

*BSI* blood stream infection, *COVID* Coronavirus disease, extended-spectrum β-lactamases (ESBLs), *XDR* extensive-drug resistance, *Ceph-R PA* cephalosporin resistant *Pseudomonas aeruginosa*, *TZP* Piperacillin-Tazobactam-producing, *CRPA* carbapenemase resistant *Pseudomonas aeruginosa*, *MRSA* methicillin-resistant *S. aureus*, *VRE* vancomycin-resistant *E. faecium*

Data are presented as absolute and relative percentage frequencies. *p* values were computed, as for qualitative variables, by the Chi-square test or the Fisher exact test, with Freeman-Halton’s extension, when appropriate. *p* values were computed on the overall population, pre-COVID vs. COVID negative (p*), pre-COVID VS. COVID positive (p**) and COVID negative vs. COVID positive (p***). In bold significant findings, in italic if suggestive (0.05 < *p* < 0.10)

**Fig. 4:**
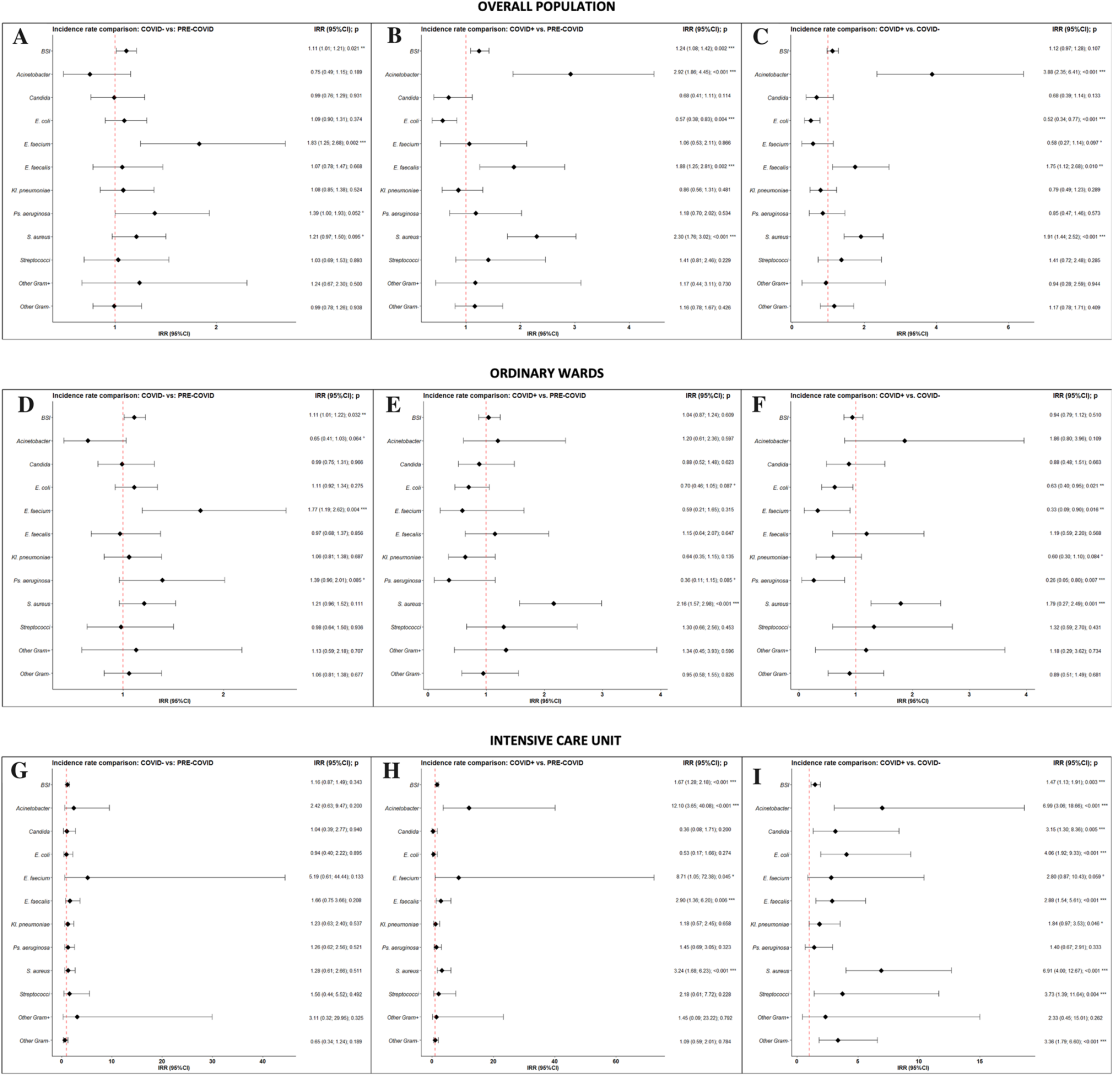
Forest Plot showing pairwise incidence rate comparisons in the overall population, ordinary wards and intensive care units. Overall population is represented in panel **A**–**C**, ordinary wards in panel **D**–**F**, and intensive care units in panels **G**–**I**. Pairwise comparisons between pre-COVID and COVID-negative cohorts are reported in panels **A**, **D** and **G**, while between pre-COVID and COVID-positive cohorts in panels **B**, **E** and **H** and between COVID-negative and COVID-positive cohorts in panels **C**, **F** and **I**. *IRR* incidence rate ratio, *95% CI* 95% confidence interval

*S. aureus* was the most incident pathogen causing BSI (Fig. 3), with 0.3 new infections per 100 patients per day (95% CI 0.21–0.32). Noteworthy, when comparing to both pre-COVID and COVID negative cohorts, *S. aureus* was significantly more incident among COVID-positive patients, and this significance was maintained in both OWs and ICUs (*p* < 0.001). MRSA accounted for 40.5% (*n* = 160) of all *S. aureus* isolates, peaking in COVID-OWs, where they caused half of the BSIs due to *S. aureus.* As shown in Fig. 4, another gram-positive bacterium that demonstrated higher IR in COVID ICUs was *E. faecalis*, showing a 3-times higher incident risk when compared to both pre-COVID (IRR 2.9, [95% CI 1.36–6.2]) and COVID negative ICUs (IRR 2.88 [95% CI 1.54–5.61]). *E. faecium*, on the opposite, resulted significantly more incidents in COVID-negative OWs. Overall rate of the VRE phenotype was 37.8%.

Similarly, we disclosed an increase in BSIs due to *Acinetobacter spp.* among COVID-positive patients, as compared to both COVID-negative (IRR 3.88 [95% CI 2.35–6.41]) and pre-COVID cohorts (IRR 2.92, [1.86–4.45]), along with a significantly higher rate of the extensively drug-resistant (XDR) phenotype (40.0% vs 11.8%, *p* = 0.008, and vs. 7.1%; *p* < 0.001, respectively). As shown in Fig. 3 and in Table S4 (see supplement), incidence rates ranged from 0.02 (95% CI 0.01–0.06) new infections per 100 patients per day in the pre-COVID ICUs to 0.24 (95% CI 0.16–0.35) in COVID ICUs (Fig. 4).

Conversely, the overall incidence of *E. coli*, the most frequently isolated pathogen in this study, was found to be significantly lower in COVID settings, except for COVID-ICUs. BSI due to *K. pneumoniae* demonstrated a similar incidence rate in the three cohorts, with an overall frequency of carbapenem-resistant strains of 29.6% (*n* = 83/2534, Table 1). Likewise, the overall incidence rate of *P. aeruginosa* was similar in the three cohorts.

## Conclusions

This real-life, observational study describes the epidemiological trend of bloodstream infections in a large cohort of patients hospitalized from 2018 to 2021, comparing incidence rates among OWs and ICUs before and during the COVID-19 pandemic. We found that, once blood cultures are performed, patients admitted to COVID settings had an overall higher risk of having BSI, with the greatest risk experienced by patients admitted to COVID-ICUs.

Overall, when compared to the pre-COVID period, COVID-positive patients had a 43% lower incident risk of developing BSI due to *E. coli*, whilst the risk of infection due to *S. aureus*, and *Acinetobacter* spp. increased, respectively, by a twofold and a threefold factor, and those differences were more pronounced in COVID-ICUs. This is concerning, especially because COVID intensive care units were the setting that demonstrated the highest prevalence of difficult-to-treat pathogens, with nearly one *S. aureus* out of two being susceptible to methicillin and only 3% of *Acinetobacter* spp. isolates being susceptible to carbapenems.

Our study provides consistent evidence that, among the pathogens causing BSI in our center, COVID-19 pandemic led to a significant shift in microbiology and AMR patterns. In particular, it is reasonable to believe that both the irrational use of personal protective equipment (inappropriate glove hygiene and gown changes) and the unprecedented workload experienced by healthcare workers [12], along with limited laboratory capacity, contributed to a diffuse loss of adherence to infection control practices [13]. Also, disruption of proper screening and isolation of patients colonized by resistant pathogens [5], along with increased exposure to antimicrobials, is likely to have promoted the selection and in-hospital diffusion of resistant phenotypes. This assumption is consistent with the fact that all infections reported in this study were hospital-acquired and is supported by the consideration that, in the early months of the pandemic, empiric broad-spectrum antibiotics were part of the recommended therapy for all critically ill COVID patients [13, 14].

A key finding of this study is that pathogens are known to colonize human skin and hospital environments—namely *S. aureus* [16] and *Acinetobacter* spp. [17]—were the ones that showed the most striking increase in relative incidence rates among COVID patients. Indeed, *S. aureus* alone was responsible for, respectively, 32.6% of all bloodstream infections diagnosed among patients admitted to COVID ordinary wards, and for 28.6% of all BSIs registered in the COVID-positive cohort (see Table S4, see supplement). In the ICU, apart from an excessive workload, other factors that may have contributed to this surge in BSI due to *S. aureus* might have been the heavy use of immunomodulatory drugs and the onset of lung dysbiosis [18, 19]. Also, in our center, admission to the COVID ICU was primarily driven by disease progression towards ARDS and, as a result, part of the increased incidence we documented in this setting is likely to be due to the use of invasive mechanical ventilation, a well-known risk factor for the incidence of bloodstream infections [19].

Noteworthy, in the pandemic period, the crude number of BSI due to *Acinetobacter* spp. was almost four times higher in patients admitted to COVID settings (Fig. 4A). This is consistent with the results of a metanalysis [20], and with experiences reported from other centers [4, 21] but, to our knowledge, this is the first study reporting incidence from both COVID-19 ordinary wards and intensive care units. In addition, this is the first study providing detailed data about resistance phenotypes.

Another peculiar finding of our work is that *E. coli*, the most frequently isolated bacteria, showed a reduced IRR in the COVID-19 general population when compared to both COVID-negative patients and patients admitted in the pre-pandemic period. A possible explanation for this phenomenon is that during COVID-19 hospitalization peaks (i.e., March–April 2020 and November 2020–January 2021) elective surgery was consistently reduced, with COVID positive patients suffering the longest delays and often being a candidate for non-urgent procedures only after swab negativization.

Our study has several limitations. On one hand, incidence has not been calculated on the overall hospital population, but only on the fraction of patients for whom at least one blood culture was ordered, thus undermining the generalizability of the results. However, comparisons between ward types and between historical periods, as well as the prevalence of resistance patterns, might be read as representative of an epidemiological shift potentially experienced by other centers during the ongoing pandemic. Also, the high sample size and the peculiar methodology of real-time data collection, involving Data Integration, Analytics and artificial intelligence, might have in part counterbalanced these limitations [9]. On the other hand, we were not able to provide data about clinical outcomes (including the likely source of infection and in-hospital death) and death, since they were not available in the Data Mart at the time the present study was conducted.

## Supplementary Information

Below is the link to the electronic supplementary material.

Supplementary file1 (DOCX 64 KB)
