# Supplementary material for: Incidence of bloodstream infections due to multidrug-resistant pathogens in ordinary wards and intensive care units before and during the COVID-19 pandemic: a real-life, retrospective observational study
Source: Infection. 2023 Mar 3;51(4):1061–9. doi: 10.1007/s15010-023-02000-3 (PMC9983510; doi:10.1007/s15010-023-02000-3)
Supplement: Supplementary file 1 — Supplementary file1 (DOCX 64 KB) [file 15010_2023_2000_MOESM1_ESM.docx]

**Incidence of multidrug-resistant bloodstream infections in ordinary wards and intensive care units before and during the COVID-19 pandemic: a real-life, retrospective observational study**

Francesco Vladimiro Segala, Pia Clara Pafundi, Carlotta Masciocchi, Barbara Fiori, Eleonora Taddei, Laura Antenucci, Giulia De Angelis, Silvia Guerriero, Roberta Pastorino, Andrea Damiani, Brunella Posteraro, Maurizio Sanguinetti, Gennaro De Pascale, Massimo Fantoni, and Rita Murri.

Supplemental material

| **­**  **Table S1.** Spectrum of organisms identified during the study period and stratified by period of onset of bloodstream infection (BSI) in ordinary wards. | | | | | | | | | | | | |
| --- | --- | --- | --- | --- | --- | --- | --- | --- | --- | --- | --- | --- |
| **Organisms** |  | **Isolates (percent of phenotypes per species) from** | | | | | | | | | | |
|  | **Total BSIs** | | **pre-COVID** | | **COVID-** | | **COVID+** | | **p^overall^** | **p^*^** | **p**** | **p***** |
|  | **No. (%)** | **%** | **No. (%)** | **%** | **No. (%)** | **%** | **No. (%)** | **%** |  |  |  |  |
| **Gram-negative bacteria** | **1129** | **52.5** | **575** | **54.4** | **486** | **52.5** | **68** | **40.0** |  |  |  |  |
| *Enterobacteriaceae* | 685 | 31.8 | 348 | 32.9 | 300 | 32.4 | 37 | 21.8 |  |  |  |  |
| *Escherichia coli* | 453 | 21.1 | 228 | 21.6 | 200 | 21.6 | 25 | 14.7 | 0.617 | 0.573 | 0.507 | 0.358 |
| 3GCeph resistant | 140 (30.9) |  | 71 (31.1) |  | 62 (31) |  | 7 (28) |  | 0.949 | 0.975 | 0.747 | 0.759 |
| *Klebsiella pneumoniae* | 232 | 10.8 | 120 | 11.4 | 100 | 10.8 | 12 | 7.1 | 0.734 | 0.986 | 0.443 | 0.443 |
| 3GCeph resistant | 52 (22.4) |  | 20 (16.7) |  | 27 (27) |  | 5 (41.7) |  | **0.049** | *0.063* | **0.035** | 0.288 |
| Carbapenem resistant | 64 (27.6) |  | 32 (26.7) |  | 29 (29) |  | 3 (25) |  | 0.909 | 0.700 | 0.901 | 0.772 |
| Nonfermenting gram-negative bacilli | 204 | 9.5 | 106 | 10.0 | 85 | 9.2 | 13 | 7.7 |  |  |  |  |
| *Acinetobacter* | 90 | 4.2 | 53 | 5.0 | 27 | 2.9 | 10 | 5.9 | **0.024** | **0.036** | 0.235 | **0.012** |
| Carbapenem resistant | 68 (75.6) |  | 39 (73.6) |  | 21 (77.8) |  | 8 (80) |  | 0.865 | 0.682 | 0.669 | 0.884 |
| XDR | 7 (7.8) |  | 4 (7.5) |  | 1 (3.7) |  | 2 (20) |  | 0.258 | 0.502 | 0.219 | 0.107 |
| *Pseudomonas aeruginosa* | 114 | 5.3 | 53 | 5.0 | 58 | 6.3 | 3 | 1.8 | *0.084* | 0.145 | 0.165 | *0.056* |
| Aminoglycoside resistant | 10 (8.8) |  | 5 (9.4) |  | 4 (6.9) |  | - |  | 0.853 | 0.881 | 0.577 | 0.596 |
| 3GCeph resistant | 20 (17.5) |  | 9 (17.0) |  | 11 (19.0) |  | - |  | 0.694 | 0.786 | 0.436 | 0.405 |
| Fluoroquinolone resistant | 23 (20.2) |  | 11 (10.4) |  | 10 (17.2) |  | 2 (66.7) |  | 0.114 | 0.637 | *0.067* | **0.036** |
| Piperacillin/tazobactam resistant | 28 (24.6) |  | 12 (11.4) |  | 15 (25.9) |  | 1 (33.3) |  | 0.868 | 0.693 | 0.670 | 0.774 |
| Carbapenem resistant | 16 (14.0) |  | 6 (11.3) |  | 9 (15.5) |  | 1 (33.3) |  | 0.508 | 0.518 | 0.262 | 0.416 |
| Ceftazidime/avibactam | 6 (5.3) |  | 3 (5.7) |  | 3 (5.2) |  | - |  | 0.912 | 0.910 | 0.672 | 0.686 |
| Ceftolozane/tazobactam | 7 (6.1) |  | 2 (3.8) |  | 5 (8.6) |  | - |  | 0.514 | 0.294 | 0.732 | 0.596 |
| Other Gram negatives | 240 | 11.2 | 121 | 11.5 | 101 | 10.9 | 18 | 10.6 | 0.791 | 0.976 | 0.503 | 0.518 |
| **Gram-positive cocci** | **970** | **45.1** | **460** | **43.6** | **412** | **44.5** | **98** | **57.6** |  |  |  |  |
| *Staphylococcus. aureus* | 337 | 15.7 | 147 | 13.9 | 140 | 15.1 | 50 | 29.4 | **<0.001** | 0.244 | **<0.001** | **<0.001** |
| MRSA | 138 (40.9) |  | 55 (37.4) |  | 58 (41.4) |  | 25 (50) |  | 0.291 | 0.487 | 0.118 | 0.294 |
| *Staphylococcus coagulase negative* | 26 | 1.2 | 14 | 1.3 | 11 | 1.2 | 1 | 0.6 | 0.857 | 0.888 | 0.579 | 0.622 |
| *Enterococcus faecium* | 107 | 5.0 | 43 | 4.1 | 60 | 6.5 | 4 | 2.3 | **0.015** | **0.008** | 0.561 | 0.101 |
| VRE | 38 (35.5) |  | 16 (37.2) |  | 21 (35) |  | 1 (25) |  | 0.881 | 0.818 | 0.627 | 0.683 |
| *Enterococcus faecalis* | 140 | 6.5 | 72 | 6.8 | 55 | 5.9 | 13 | 7.6 | 0.343 | 0.635 | 0.225 | 0.143 |
| *Streptococcus spp* | 97 | 4.5 | 49 | 4.6 | 38 | 4.1 | 10 | 5.9 | 0.280 | 0.747 | 0.158 | 0.114 |
| Other Gram Positives | 40 | 1.9 | 19 | 1.8 | 17 | 1.8 | 4 | 2.3 | 0.638 | 0.825 | 0.343 | 0.423 |
| **Fungi** |  |  |  |  |  |  |  |  |  |  |  |  |
| *Candida spp* | 223 | 10.4 | 116 | 11.0 | 91 | 9.8 | 16 | 9.4 | 0.826 | 0.676 | 0.728 | 0.577 |
| **Anaerobic** | 52 | 2.4 | 21 | 2.0 | 27 | 2.9 | 4 | **2.3** | 0.301 | 0.130 | 0.443 | 0.968 |
| **Total organisms** | **2151** |  | **1056** |  | **925** |  | **170** |  |  |  |  |  |
| **Abbreviations**: BSI: Bloodstream Infection; COVID: Coronavirus disease; extended-spectrum β-lactamases (ESBLs); XDR: extensive-drug resistance; Ceph-R PA: cephalosporin resistant *Pseudomonas aeruginosa*; TZP: Piperacillin-Tazobactam-producing; CRPA: carbapenemase resistant *Pseudomonas aeruginosa*; MRSA methicillin-resistant *S. aureus*, VRE vancomycin-resistant *E. faecium.*  ^§^ Data are presented as absolute and relative percentage frequencies. p-values were computed, as for qualitative variables, by the Chi-square test or the Fisher exact test, with Freeman-Halton’s extension, when appropriate. p-values were computed on overall population, pre-COVID vs. COVID- (p*), pre-COVID VS. COVID+ (p**) and COVID- vs. COVID+ (p***). In bold significant findings, in italic if suggestive (0.05<p<0.10). | | | | | | | | | | | | |

| **Table S2.** Spectrum of organisms identified during the study period and stratified by period of onset of bloodstream infection (BSI) in ICU. | | | | | | | | | | | | |
| --- | --- | --- | --- | --- | --- | --- | --- | --- | --- | --- | --- | --- |
| **Organisms** |  | **Isolates (percent of phenotypes per species) from** | | | | | | | | | | |
|  | **Total BSIs** | | **pre-COVID** | | **COVID-** | | **COVID+** | | **p^overall^** | **p^*^** | **p**** | **p***** |
|  | **No. (%)** | **%** | **No. (%)** | **%** | **No. (%)** | **%** | **No. (%)** | **%** |  |  |  |  |
| **Gram-negative bacteria** | **210** | **54.8** | **68** | **61.3** | **68** | **52.3** | **74** | **52.1** |  |  |  |  |
| *Enterobacteriaceae* | 73 | 19.1 | 27 | 24.3 | 29 | 22.3 | 17 | 12.0 |  |  |  |  |
| *Escherichia coli* | 25 | 6.5 | 11 | 9.9 | 10 | 7.7 | 4 | 2.8 | 0.663 | 0.636 | 0.371 | 0.599 |
| 3GCeph resistant | 15 (60) |  | 7 (63.4) |  | 5 (50) |  | 3 (75) |  | 0.653 | 0.528 | 0.680 | 0.393 |
| *Klebsiella pneumoniae* | 48 | 12.5 | 16 | 14.4 | 19 | 14.6 | 13 | 9.2 | 0.709 | 0.852 | 0.426 | 0.516 |
| 3GCeph resistant | 11 (22.9) |  | 5 (31.2) |  | 6 (31.6) |  | - |  | *0.071* | 0.983 | **0.027** | **0.025** |
| Carbapenem resistant | 19 (39.6) |  | 5 (31.2) |  | 7 (36.8) |  | 7 (53.8) |  | 0.443 | 0.728 | 0.219 | 0.341 |
| Nonfermenting gram-negative bacilli | 80 | 20.9 | 17 | 15.3 | 24 | 18.5 | 39 | 27.5 |  |  |  |  |
| *Acinetobacter* | 35 | 9.1 | 3 | 2.7 | 7 | 5.4 | 25 | 17.6 | **<0.001** | 0.272 | **<0.001** | **<0.001** |
| Carbapenem resistant | 18 (57.1) |  | 2 (66.7) |  | 4 (57.1) |  | 12 (48) |  | 0.783 | 0.778 | 0.541 | 0.669 |
| XDR | 15 (42.9) |  | - |  | 3 (42.9) |  | 12 (48) |  | 0.284 | 0.175 | 0.112 | 0.810 |
| *Pseudomonas Aeruginosa* | 45 | 11.7 | 14 | 12.6 | 17 | 13.1 | 14 | 9.9 | 0.345 | 0.812 | 0.175 | 0.240 |
| Amino | - |  | - |  | - |  | - |  | n.a. | n.a. | n.a. | n.a. |
| Ceph-R PA | 7 (15.6) |  | 1 (7.1) |  | 4 (23.5) |  | 2 (14.2) |  | 0.451 | 0.217 | 0.541 | 0.517 |
| Fluoroquinolones | 3 (6.7) |  | - |  | 2 (11.8) |  | 1 (7.1) |  | 0.424 | 0.185 | 0.309 | 0.665 |
| TZP-producing | 10 (22.2) |  | 4 (28.6) |  | 4 (23.5) |  | 2 (14.2) |  | 0.653 | 0.750 | 0.357 | 0.517 |
| CRPA | 6 (13.3) |  | 1 (7.1) |  | 4 (23.5) |  | 1 (7.1) |  | 0.293 | 0.217 | 1.000 | 0.217 |
| Ceftazidime/avibactam | - |  | - |  | - |  | - |  | n.a. | n.a. | n.a. | n.a. |
| Ceftolozane/tazobactam | - |  | - |  | - |  | - |  | n.a. | n.a. | n.a. | n.a. |
| Other Gram negatives | 57 | 14.9 | 24 | 21.6 | 15 | 11.5 | 18 | 12.7 | *0.053* | *0.069* | 0.487 | **0.018** |
| **Gram-positive cocci** | **161** | **42.0** | **39** | **35.1** | **57** | **43.8** | **65** | **45.8** |  |  |  |  |
| *Staphylococcus aureus* | 58 | 15.1 | 13 | 11.7 | 16 | 12.3 | 29 | 20.4 | **<0.001** | 0.790 | **<0.001** | **<0.001** |
| MRSA | 22 (37.9) |  | 3 (23.1) |  | 6 (37.5) |  | 13 (44.8) |  | 0.405 | 0.404 | 0.180 | 0.634 |
| *Staphylococcus coagulase negative* | 6 | 1.7 | 2 | 1.8 | 3 | 2.3 | 1 | 0.7 | 0.857 | 0.744 | 0.874 | 0.668 |
| *Enterococcus faecium* | 12 | 3.1 | 1 | 0.9 | 5 | 3.8 | 6 | 4.2 | 0.033 | 0.132 | **0.009** | 0.178 |
| VRE | 7 (58.3) |  | - |  | 2 (40) |  | 5 (83.3) |  | 0.162 | 0.439 | *0.088* | 0.137 |
| *Enterococcus. faecalis* | 46 | 12.0 | 10 | 9.0 | 16 | 12.3 | 20 | 14.1 | **0.002** | 0.363 | **0.001** | **0.009** |
| Streptococci | 16 | 4.2 | 4 | 3.6 | 6 | 4.6 | 6 | 4.2 | 0.312 | 0.644 | 0.145 | 0.281 |
| Other Gram Positives | 5 | 1.3 | 1 | 0.9 | 3 | 2.3 | 1 | 0.7 | 0.658 | 0.372 | 0.721 | 0.668 |
| **Fungi** |  |  |  |  |  |  |  |  |  |  |  |  |
| *Candida spp* | 18 | 4.7 | 8 | 7.2 | 8 | 6.1 | 2 | 1.4 | 0.504 | 0.825 | 0.244 | 0.311 |
| **Anaerobic** | **12** | **3.1** | **4** | **3.6** | **5** | **3.8** | **3** | **2.1** | 0.961 | 0.865 | 0.781 | 0.893 |
| **Total organisms** | **383** |  | **111** |  | **130** |  | **142** |  |  |  |  |  |
| **Abbreviations**: BSI: Blood Stream Infection; COVID: Coronavirus disease; extended-spectrum β-lactamases (ESBLs); XDR: extensive-drug resistance; Ceph-R PA: cephalosporin resistant *Pseudomonas aeruginosa*; TZP: Piperacillin-Tazobactam-producing; CRPA: carbapenemase resistant *Pseudomonas aeruginosa*; MRSA methicillin-resistant *S. aureus*, VRE vancomycin-resistant *E. faecium.*  ^§^ Data are presented as absolute and relative percentage frequencies. p-values were computed, as for qualitative variables, by the Chi-square test or the Fisher exact test, with Freeman-Halton’s extension, when appropriate. p-values were computed on overall population, pre-COVID vs. COVID- (p*), pre-COVID VS. COVID+ (p**) and COVID- vs. COVID+ (p***). In bold significant findings, in italic if suggestive (0.05<p<0.10). | | | | | | | | | | | | |

**Table S3.** Definition of resistance phenotypes

| Microorganism | Classification | Definition |
| --- | --- | --- |
| *Staphylococcus aureus* | MRSA | Resistant to oxacillin |
| *Enterococcus faecium* | VRE | Resistant to vancomycin |
| *Escherichia coli* | 3GCeph resistant | Resistant to cefotaxime **OR** ceftazidime **OR** cefepime **AND** susceptible to meropenem |
| *Klebsiella pneumoniae* | 3GCeph resistant | Resistant to cefotaxime **OR** ceftazidime **OR** cefepime **AND** susceptible to meropenem |
|  | Carbapenem resistant | Resistant to meropenem |
| *Acinetobacter spp* | Carbapenem resistant | Resistant to meropenem |
|  | XDR | Resistant to meropenem **AND** colistin |
| *Pseudomonas aeruginosa* | Aminoglycoside resistant | Resistant to amikacin **OR** gentamicin (2018-2019)  Resistant to amikacin **OR** tobramycin (2020)  Resistant to amikacin (2021) |
|  | 3GCeph resistant | Resistant to cefepime **OR** ceftazidime |
|  | Fluoroquinolone resistant | Resistant to ciprofloxacin |
|  | Piperacillin/tazobactam resistant | Resistant to piperacillina/tazobactam |
|  | Carbapenem resistant | Resistant to imipenem **OR** meropenem |

| **Table S4.** Incidence rate per 100 persons-days of hospitalization, both on the overall population and on ordinary wards and ICU, stratified for study subgroups (N=14,884). | | | | | | | | | | | |
| --- | --- | --- | --- | --- | --- | --- | --- | --- | --- | --- | --- |
|  | **Pre-COVID** | | | **COVID –** | | | **COVID +** | | | | |
| **Overall**  **(N=14,884)** | **(N=7,502)**  **Person-days: 143,316** | | | **(N=6,299)**  **Person-days: 115,772** | | | **(N=1,203)**  **Person-days: 30,708** | | | | |
|  | **N** | **IR** | **95%CI** | **N** | **IR** | **95%CI** | **N** | **IR** | | | **95%CI** |
| Positive BSI | 1038 | 0.72 | 0.68; 0.77 | 931 | 0.80 | 0.75; 0.86 | 276 | 0.90 | | | 0.80; 1.01 |
| *Acinetobacter* | 56 | 0.04 | 0.03; 0.05 | 34 | 0.03 | 0.02; 0.04 | 35 | 0.11 | | | 0.08; 0.16 |
| *E. coli* | 239 | 0.17 | 0.15; 0.19 | 210 | 0.18 | 0.16; 0.21 | 29 | 0.09 | | | 0.07; 0.14 |
| *E. faecium* | 44 | 0.03 | 0.02; 0.04 | 65 | 0.06 | 0.04; 0.07 | 10 | 0.03 | | | 0.02; 0.06 |
| *E. faecalis* | 82 | 0.06 | 0.05; 0.07 | 71 | 0.06 | 0.05; 0.08 | 33 | 0.11 | | | 0.08; 0.15 |
| *K. pneumoniae* | 136 | 0.09 | 0.08; 0.11 | 119 | 0.10 | 0.09; 0.12 | 25 | 0.08 | | | 0.05; 0.12 |
| *P. aeruginosa* | 67 | 0.05 | 0.04; 0.06 | 75 | 0.07 | 0.05; 0.08 | 17 | 0.05 | | | 0.03; 0.09 |
| *S. aureus* | 160 | 0.11 | 0.10; 0.13 | 156 | 0.13 | 0.11; 0.16 | 79 | 0.30 | | | 0.21; 0.32 |
| *Streptococcus spp* | 53 | 0.04 | 0.03; 0.05 | 44 | 0.04 | 0.03; 0.05 | 16 | 0.05 | | | 0.03; 0.08 |
| Other Gram+ | 20 | 0.01 | 0.01; 0.02 | 20 | 0.02 | 0.01; 0.03 | 5 | 0.02 | | | 0.01; 0.04 |
| Other Gram- | 152 | 0.10 | 0.09; 0.12 | 116 | 0.10 | 0.08; 0.12 | 36 | 0.12 | | | 0.08; 0.16 |
| *Candida spp* | 124 | 0.09 | 0.07; 0.10 | 99 | 0.09 | 0.07; 0.10 | 18 | 0.06 | | | 0.04; 0.09 |
| **Ordinary Wards**  **(N=13,332)** | **(N=6,812)**  **Person-days: 127,940** | | | **(N=5,663)**  **Person-days: 100,964** | | | **(N=857)**  **Person-days: 20,121** | | | | |
|  | **N** | **IR** | **95%CI** | **N** | **IR** | **95%CI** | **N** | **IR** | | **95%CI** | |
| Positive BSI | 931 | 0.73 | 0.68; 0.78 | 814 | 0.81 | 0.75; 0.86 | 153 | 0.76 | | 0.65; 0.89 | |
| *Acinetobacter* | 53 | 0.04 | 0.03; 0.05 | 27 | 0.03 | 0.02; 0.04 | 10 | 0.05 | | 0.03; 0.09 | |
| *E. coli* | 228 | 0.18 | 0.16; 0.20 | 200 | 0.20 | 0.17; 0.23 | 25 | 0.12 | | 0.08; 0.18 | |
| *E. faecium* | 43 | 0.03 | 0.02; 0.04 | 60 | 0.06 | 0.05; 0.08 | 4 | 0.02 | | 0.01; 0.05 | |
| *E. faecalis* | 72 | 0.06 | 0.04; 0.07 | 55 | 0.05 | 0.04; 0.07 | 13 | 0.06 | | 0.04; 0.11 | |
| *K. pneumoniae* | 120 | 0.09 | 0.08; 0.11 | 100 | 0.10 | 0.08; 0.12 | 12 | 0.06 | | 0.03; 0.10 | |
| *P. aeruginosa* | 53 | 0.04 | 0.03; 0.05 | 58 | 0.06 | 0.04; 0.07 | 3 | 0.01 | | 0.00; 0.05 | |
| *S. aureus* | 147 | 0.12 | 0.10; 0.13 | 140 | 0.14 | 0.12; 0.16 | 50 | 0.25 | | 0.19; 0.33 | |
| *Streptococcus spp* | 49 | 0.04 | 0.03; 0.05 | 38 | 0.04 | 0.03; 0.05 | 10 | 0.05 | | 0.03; 0.09 | |
| Other Gram+ | 19 | 0.01 | 0.01; 0.02 | 17 | 0.02 | 0.01; 0.03 | 4 | 0.02 | | 0.01; 0.05 | |
| Other Gram- | 121 | 0.09 | 0.08; 0.11 | 101 | 0.10 | 0.08; 0.12 | 18 | 0.09 | | 0.06; 0.14 | |
| *Candida spp* | 116 | 0.09 | 0.08; 0.11 | 91 | 0.09 | 0.07; 0.11 | 16 | 0.08 | | 0.05; 0.13 | |
| **ICU**  **(N=1,552)** | **(N=570)**  **Person-days: 15,376** | | | **(N=636)**  **Person-days: 14,808** | | | **(N=346)**  **Person-days: 10,587** | | | | |
|  | **N** | **IR** | **95%CI** | **N** | **IR** | **95%CI** | **N** | | **IR** | | **95%CI** |
| Positive BSI | 107 | 0.70 | 0.58; 0.84 | 117 | 0.79 | 0.66; 0.95 | 123 | | 1.16 | | 0.97; 1.39 |
| *Acinetobacter* | 3 | 0.02 | 0.01; 0.06 | 7 | 0.05 | 0.02; 0.10 | 25 | | 0.24 | | 0.16; 0.35 |
| *E. coli* | 11 | 0.07 | 0.04; 0.13 | 10 | 0.07 | 0.04; 0.13 | 4 | | 0.04 | | 0.01; 0.10 |
| *E. faecium* | 1 | 0.01 | 0.00; 0.05 | 5 | 0.03 | 0.01; 0.08 | 6 | | 0.06 | | 0.02; 0.13 |
| *E. faecalis* | 10 | 0.06 | 0.03; 0.12 | 16 | 0.11 | 0.07; 0.18 | 20 | | 0.19 | | 0.12; 0.29 |
| *K. pneumoniae* | 16 | 0.10 | 0.06; 0.17 | 19 | 0.13 | 0.08; 0.20 | 13 | | 0.12 | | 0.07; 0.21 |
| *P. aeruginosa* | 14 | 0.09 | 0.05; 0.15 | 17 | 0.11 | 0.07; 0.18 | 14 | | 0.13 | | 0.08; 0.22 |
| *S. aureus* | 13 | 0.08 | 0.05; 0.15 | 16 | 0.11 | 0.07; 0.18 | 29 | | 0.27 | | 0.19; 0.39 |
| *Streptococcus spp* | 4 | 0.03 | 0.01; 0.07 | 6 | 0.04 | 0.02; 0.09 | 6 | | 0.06 | | 0.02; 0.13 |
| Other Gram+ | 1 | 0.01 | 0.00; 0.05 | 3 | 0.02 | 0.01; 0.06 | 1 | | 0.01 | | 0.00; 0.07 |
| Other Gram- | 24 | 0.16 | 0.10; 0.23 | 15 | 0.10 | 0.06; 0.17 | 18 | | 0.17 | | 0.11; 0.27 |
| *Candida spp* | 8 | 0.05 | 0.03; 0.10 | 8 | 0.05 | 0.03; 0.11 | 2 | | 0.02 | | 0.00; 0.08 |
| **Abbreviations**: ICU: Intensive Care Unit; BSI: Blood Stream Infection; COVID: COronaVIrus Disease; IRR: Incidence Rate Ratio; 95%CI: 95% Confidence Interval | | | | | | | | | | | |

**Table S5.** Microorganisms included in the study.

| **Microorganisms’ Class** | **Pathogens** | |
| --- | --- | --- |
| **Gram+** | | |
| *Staphylococcus aureus* | Staphylococcus aureus complex | staaur |
| *Enterococcus faecium* | Enterococcus faecium (Str.faecium) | entfac |
|  | Enterococcus faecium | strfac |
| *Enterococcus faecalis* | Enterococcus faecalis (Str.faecalis) | entfae |
|  | Enterococcus faecalis | strfae |
| **Gram-** | | |
| *Escherichia coli* | Escherichia coli | esccol |
| *Kebsiellal pneumoniae* | Klebsiella pneumoniae | klepn |
|  | Klebsiella pneumoniae | kleppt |
|  | Klebsiella pneumoniae | klpnsp |
| *Pseudomonas aeruginosa* | Pseudomonas aeruginosa | pseaer |
| *Acinetobacter spp.* | Acinetobacter baumannii/haemol | acibah |
|  | Acinetobacter baumannii | acibau |
|  | Acinetobacter baumannii/calcoacetic | acibcg |
|  | Acinetobacter haemolyticus | acihae |
|  | Acinetobacter lwoffii | acilwo |
|  | Acinetobacter pittii | acipi |
|  | Acinetobacter spp | acispe |
|  | Acinetobacter radioresistans | acrad |
| *Enterobacter* *spp* | Enterobacter kobei | enko |
|  | Enterobacter agglomerans | entag |
|  | Enterobacter asburiae | entasb |
|  | Enterobacter cloacae | entclo |
|  | Enterobacter gergoviae | entger |
|  | Enterobacter spp | entspe |
| *Serratia* *spp.* | Serratia liquefaciens | serliq |
|  | Serratia marcescens | sermar |
|  | Serratia rubidaea | serrub |
| *Proteus* *spp.* | Proteus mirabilis | promir |
|  | Proteus vulgaris | provul |
|  | Proteus spp. | prspp |
| **Fungi** | | |
| *Candida* *spp.* | Candida albicans | canalb |
|  | Candida glabrata | cangla |
|  | Candida guilliermondii | cangui |
|  | Candida krusei | cankru |
|  | Candida parapsilosis | canpar |
|  | Candida tropicalis | cantro |
